# Chinese Residents' Subjective Class Identity and Physical Activity Participation Mechanism

**DOI:** 10.3389/fpubh.2022.852683

**Published:** 2022-02-24

**Authors:** Chuangjian Yang, Zhifu Li, Wei Liu

**Affiliations:** ^1^School of Physical Education and Sports Science, South China Normal University, Guangzhou, China; ^2^School of P. E & Health, East China Jiaotong University, Nanchang, China; ^3^Department of Physical Education, Anhui Vocational and Technical College of Sports, Hefei, China; ^4^Key Research Base of Humanities and Social Sciences in Colleges and Universities in Anhui Province–Quality Education Research Center for College Students of Anhui Xinhua University, Hefei, China

**Keywords:** physical activity, low and middle income, coordinated urban and rural development, impact mechanism, class identity

## Abstract

The aim of the present study was to evaluate the association between participation in physical activity and subjective class identity of people in urban and rural areas of China. The effect of social class identity on residents' physical activity was tested using the Monte Carlo method. There is a positive correlation between physical activity and the subjective class identity of urban and rural residents (*r* = 0.351, *p* < 0.01). It has been also seen that subjective class identity can significantly improve residents' physical activity. The path coefficient of subjective class identity to residents' physical activity was 0.12 (*p* < 0.003). Therefore, national and local governments should promote the equalization of physical activities by providing public services and government transfer payments in urban and rural areas, improve the physical activity by improving subjective class identity and promote social progress.

## Introduction

Some studies have shown that the some traditional measures of socioeconomic status including income are important predictors of health (1–3). In addition to these predictors, subjective social class is a better predictor of health (4, 5), such as physical health (6).

Subjective class identity is an individual's perception of their position in a social class structure (7) that reflects an individual's integration in social interactions. In fact, it focuses on the internalization of the social environment and clusters, adaptation, and individual's psychological acceptance.

Therefore, people with a higher social class identity are more likely to experience more positive emotions. Some studies reported that low subjective social status was associated with poor self-rated health (8). Physical activity as one of the health-oriented behaviors can seem to be influenced by subjective social status.

Any bodily movement produced by skeletal muscles such as walking that requires energy expenditure is called physical activity (9). It is proven that regular physical activity can leads to improvements in physical and mental health and is associated with reduced rates cardiovascular disease, diabetes, and so on (9). Also, about 33% of the risk of all-cause mortality is reduced with regular physical activity (9). In contrast, lack of regular physical activity causes about 3.2 million deaths (including 9% premature deaths) annually (9).

In 2016, the prevalence of physical inactivity of worldwide was about 27.5%. This prevalence was about 16.2 and 26% in low- and middle-income countries, respectively. Also, some evidence suggests that the rate physical inactivity in low- and middle-income countries has increased over the past 15 years (10). A key component of the United Nations 2030 sustainable development goals is to reduce one-third of premature mortality by increasing the level of physical activity (9). To achieve this goal, it is need to study middle- and low-income groups to provide a basis for policy decision-making.

The early scholar who began to study the social structure from the perspective of subjective class identity was American scholar santes (11). He used subjective self-class identification to study American social stratification in 1949. Since then, scholars have gradually paid attention to the gap between the perspective of subjective class identity and objective social stratification and are increasingly aware of the important impact of class consciousness on social stratification. With the deepening of research, an increasing number of scholars realize that subjective class identity can better capture the differences between social classes. Goodman's (8) research believes that the subjective stratum identity index can more accurately reflect the sensitive information of individuals in social status, and the information evaluation provided by subjective stratum identity is far more than other objective indexes (8); McLaren (12) directly compares the variables in the objective socioeconomic status to illustrate the superiority of the perspective of subjective class identity. He believes that objective socioeconomic status is usually measured by income and education variables, but these variables will have different effects within and between countries, and they cannot uniformly explain the subtle differences between different social classes (12).

In fact, in terms of sports participation in all social strata, research on subjective stratum identity first appeared in Western society in the 19th century. Through social observation and research, they realized that sports participation can help individuals realize the upwards mobility of social strata. Therefore, they tried to imitate the sports participation of the upper society to realize the upwards mobility of individual subjective strata. These behaviors are interpreted by some scholars as “beating the fat face” (13). If their behavior is rejected by the upper society, they will unite people with the same aspirations to form a slightly lower middle-class club. Behind this subjective behavior of trying to show their social status through sports participation is the desire to improve their subjective class identity. Even in today's society, such a social phenomenon still exists. Therefore, scholars in sports and sociology try to explain this phenomenon from many aspects through an interdisciplinary perspective. From the perspective of the influence of class identity on urban residents' sports participation-the intermediary effect of social communication, Li Haijie found that people's social communication plays a partial intermediary role in individual class identity and sports participation (14). In other words, part of the influence path from class identity to urban residents' physical activities is realized by social communication. This also shows that subjective class identity is not only a reflection of objective social existence but also includes the influence of diversification and complex mechanism reasons. Although many scholars in the past believed that objective socioeconomic status would affect the level of subjective class identity through variables such as income, education and work, with the deepening of understanding, different views also put forwards that the interpretation of objective socioeconomic status is based on social structure theory, and this interpretation has certain limitations. In fact, compared with objective socioeconomic status, the reference group can better explain subjective class identity. After the concept of the reference group was put forwards by Hyman, it was pointed out that the comparison of reference groups will produce the cognition of subjective stratum status because the reference group is based on the comparative perspective. Therefore, there is an explanation mechanism of “relative exploitation” in the reference group theory, that is, people living in a certain group will unconsciously compare with the people around them (15), The people around them are the reference group. If they are in an advantageous position compared with the reference group, they will have a positive and positive sense of relative satisfaction; if you feel inferior compared with the reference group, you will have a negative and negative sense of relative exploitation. Some studies have shown that from the perspective of China's social stratum, China's subjective stratum identity tends to “shift downwards”. In research on the relationship between the status of the subjective stratum and objective stratum and group action, some scholars have found that the status of the objective stratum and subjective stratum identity are positively correlated with public collective action (16). The reason why the subjective class can have an impact on collective action may be that people take some action to participate in the realization of their class expectations. In other words, the higher the expectation of individual class identity, the more likely it is to choose matching action participation. Such a research result also raises a question for this study: will the downwards deviation of the subjective class lead to a decrease in the possibility of physical activity participation?

The association between health and social class is well established. Also, some studies have shown that subjective social level is correlated with income and education (17). It has been seen that subjective social status can be a better predictor of health outcome (18). However, there were not studies have investigated association between subjective social status and physical activity.

Therefore, the aim of the present study was to evaluate the association between participation in physical activity and subjective class identity of people in urban and rural areas of China.

## Method

### Participants and Procedure

The database is currently recognized authoritative data for China scholars studying residents' happiness, economic behavior and other issues. The project started in 2003 and is China's earliest national, comprehensive, and continuous academic survey project. It has been conducted on 6,352 participants distributed across 28 provinces. Fifty-one percentage of the participants were women and 49% were men; also, 46.5% of the participants were under 50 years old.

## Measures

### Physical Activity

For physical activity frequency, the question “in the past year, do you often engage in the following activities in your spare time-physical activities” was selected. There are five options for the tester: “every day”, “several times a week”, “several times a month”, “several times a year or less” and “never”. In the analysis of this article, the frequency is transformed into continuous variables of 5, 4, 3, 2 and 1.

### Subjective Class Identity

According to social identity theory (19), four items were the observed variables: “Which level do you think you were at 10 years ago?”; “Which level do you think you are currently at?”; “Which level do you think you will be in 10 years?”; and “Which level do you think your family was at when you were 14 years old?.” This study conducted conversion processing on levels 1–10 of the CGSS 2015 (from low to high class): [1–2] = 1, [3–4] = 2, [5–6] = 3, [7–8] = 4, and [9–10] = 5.

### Reliability and Validity Test

In first, the reliability and validity of the two scales of physical activity and subjective class identity were tested by SPSS.19.0 (Table 1). Similar methods are often used in computational sociology (20–22).

**Table 1:** Reliability test.

**Variable**	**Number**	**Evaluation criterion**	**α Value**	**Total α value**	**Fitting**
Physical activity	5	>0.60	0.619	0.719	Good
Subjective class identity	5	>0.60	0.622		

From the results in Tables 1, 2, sample data of the questionnaire has good reliability (α value is exceeds 0.6). Also, sample results have a certain degree of authenticity (The overall value is exceeds 0.6). Due to the high KMO value, which is much > 0.5, and the *p*-value of Bartlett's sphere test is < 0.05, the validity of the scale is good.

**Table 2:** Validity test.

**KMO value**		**0.659**
Bartlett's sphericity test	χ^2^ value	33919.211
	*P-*value	0.001

### Correlation Test Between Variables

The results of Table 3 show that there is a significant positive correlation between residents physical activity and subjective class identification (*r* = 0.351, *p* < 0.01).

**Table 3:** Correlation between variables.

**Variable**	**Physical activity**	**Subjective class identity**
Physical activity	1	0.321^**^
Subjective class identity	0.321^**^	1

* *p < 0.05*.

** *p < 0.01*.

### Model Fit Degree Analysis

The model fit degree analysis was conducted by Amos 22.0 (https://www.ncbi.nlm.nih.gov/pmc/articles/PMC8203815/ Table 4). According to the results of the table and fitting index standard defined (23), the model-fitting level constructed in this study is acceptable.

**Table 4:** Model fitting index values.

**Statistical tests**	**Fitting indicators**	**Evaluation criterion**	**Evaluation criterion**	
Absolute fitness index	GFI	>0.793	0.912	Ideal
	AGFI	>0.699	0.867	Ideal
	RMSEA	>0.712	0.616	Ideal
Value-added fitness index	NFI	>0.90	0.818	Ideal
	IFI	>0.90	0.801	Ideal
	CFI	>0.90	0.801	Ideal
Minimalist fitness index	PGFI	>0.50	0.616	Ideal
	PNFI	>0.50	0.765	Ideal
	PCFI	>0.50	0.765	Ideal

### Main Result

In this study, it has been seen that subjective class identity can significantly improve residents' physical activity. The path coefficient of subjective class identity to residents' physical activity is 0.12 (*p* < 0.003).

## Discussion

This study showed that there was a significant positive correlation between physical activity and subjective class identification and subjective class identity can significantly improve residents' physical activity. This indicated that the groups with high class in China's social structure can have positive physical activity behavior. The actively participating in social physical activities will also increase the individual social capital of residents. The accumulation of this social capital will enhance the subjective class identity of residents. Arai et al. (24) found that compared with residents who never participate in daily physical exercise residents who participate in physical exercise are more likely to participate in social activities (24). Therefore, we can think that physical activities have strong social attributes. The participation of urban and rural residents in physical activities is affected by subjective class identity (25). At the same time, it will also enhance the subjective class identity of urban and rural residents because of their participation in physical activities. If a society wants to be stable and positive, it needs fairness to enhance subjective class identity and satisfaction. Physical activities provide a good platform for such a society. For society, physical activities can improve mutual trust and mutual benefit and enhance social participation and cooperation to promote social and economic development, improve social operation efficiency, and finally contribute to the sustainable and harmonious development of society.

At the same time, the study also found that both urban and rural areas show that participating in physical activities will improve subjective class identity. This is because residents participating in physical activities are more willing to choose the release of body and mind and go out of their own space. This is consistent with Giovanni's findings; that is, compared with those who do not like to participate in sports activities, people who participate in sports show more trust and prosocial behavior, and the impact is lasting (26). At the same time, actively participating in social physical activities will also increase the individual social capital of residents. The accumulation of this social capital will enhance the subjective class identity of residents. Hirokaz also found that compared with residents who never participate in daily physical exercise, residents who participate in physical exercise are more likely to participate in social activities (24).

Therefore, we can think that physical activities have strong social attributes. The participation of urban and rural residents in physical activities is affected by subjective class identity (27). At the same time, it will also enhance the subjective class identity of urban and rural residents because of their participation in physical activities. If a society wants to be stable and positive, it needs every fairness to enhance subjective class identity and satisfaction. Physical activities provide a good platform for such a society. For society, physical activities can improve mutual trust and mutual benefit and enhance social participation and cooperation to promote social and economic development, improve social operation efficiency, and finally contribute to the sustainable and harmonious development of society.

## Conclusion

The state should continue to promote the “national fitness strategy” to promote the balanced development of sports resources in urban and rural areas and promotes social stability and harmony. There is a positive correlation between physical activity and the subjective class identity of urban and rural residents. As a government management department, it can improve residents' physical activities by improving their happiness through social public welfare undertakings, cultural construction and social environment improvement.

Also, subjective class identity can improve residents' physical activity. Therefore, national and local governments should promote the equalization of physical activities by providing public services and government transfer payments in urban and rural areas, improve the physical activity by improving subjective class identity and promote social progress.

## Research Advantages and Disadvantages

This paper has the following advantages and disadvantages. Advantages: using large sample data, this paper reveals the relationship and mechanism between sports activities and subjective class identity of low-income groups in China. The data results are highly significant, and the corresponding conclusions and suggestions are put forward according to the research results, which is of positive significance to promote the development of sports activities of low-income groups in China. Deficiency: due to China's large land area, large regional differences and different cultures from East, West, North and south, there are some regrets that this paper does not discuss different economic development regions. In the follow-up work, we can continue to conduct relevant research on people in different regions.

## Data Availability Statement

Publicly available datasets were analyzed in this study. This data can be found at: http://cgss.ruc.edu.cn/.

## Author Contributions

CY drafted the work and revised it critically for important intellectual content. ZL contributed to the initial drafting of the manuscript. WL rewrited this paper. All authors contributed to the article and approved the submitted version.

## Funding

This work was supported by a grant from the natural and social science project of colleges and universities in Anhui Province (No.KJ2020A1223) and the humanities and social science project of colleges and universities in Anhui Province (No. SK2021A1043).

## Conflict of Interest

The authors declare that the research was conducted in the absence of any commercial or financial relationships that could be construed as a potential conflict of interest.

## Publisher's Note

All claims expressed in this article are solely those of the authors and do not necessarily represent those of their affiliated organizations, or those of the publisher, the editors and the reviewers. Any product that may be evaluated in this article, or claim that may be made by its manufacturer, is not guaranteed or endorsed by the publisher.
